# Biruni Index as a Novel Diagnostic Tool for the Early Prediction of Mortality in Critical Patients With COVID-19: A Cohort Study

**DOI:** 10.7759/cureus.30705

**Published:** 2022-10-26

**Authors:** Suna Koc, Murat Deveci, Huseyin Kayadibi, Mehmet Dokur, Ismail Yildiz, Ilke Kupeli, Baris Yilmaz

**Affiliations:** 1 Department of Anesthesiology and Reanimation, Biruni University Medical Faculty, Istanbul, TUR; 2 Department of Gastroenterology, Biruni University Medical Faculty, Istanbul, TUR; 3 Department of Medical Biochemistry, Eskisehir Osmangazi University Medical Faculty, Eskisehir, TUR; 4 Department of Emergency Medicine, Biruni University Medical Faculty, Istanbul, TUR

**Keywords:** covid-19, biruni index, prognostic factors, mortality, intensive care unit

## Abstract

Background: The aim of this study was to find out the potential risk factors associated with mortality in severe coronavirus disease 2019 (COVID-19) patients hospitalized due to viral bronchopneumonia, and to establish a novel COVID-19 mortality index for daily use.

Methods: The study included 431 quantitative real-time polymerase chain reaction (qRT-PCR)-confirmed COVID-19-positive patients admitted to the intensive care unit in a tertiary care hospital. Patients were divided into training and validation cohorts at random (n= 285 and n= 130, respectively). Biruni Index was developed by multivariate logistic regression analysis for predicting COVID-19-related mortality.

Results: In univariate logistic regression analysis, age, systolic and diastolic blood pressures, respiratory and pulse rates per minute, D-dimer, pH, urea, ferritin, and lactate dehydrogenase levels at first admission were statistically significant factors for the prediction of mortality in the training cohort. By using multivariate logistic regression analysis, all of these statistically significant parameters were used to produce Biruni Index. Statistically significant differences in Biruni Index were observed between ex and non-ex groups in both training and validation cohorts (*P *< 0.001 for both comparisons). Areas under receiver operating characteristic (ROC) curve for Biruni Index were 0.901 (95CI%: 0.864-0.938, *P *< 0.001) and 0.860 (95CI%: 0.795-0.926, *P *< 0.001) in training and validation cohorts, respectively.

Conclusion: As a pioneering clinical study, Biruni Index may be a useful diagnostic tool for clinicians to predict the mortality in critically ill patients with COVID-19 hospitalized due to severe viral bronchopneumonia. However, Biruni Index should be validated with larger series of multicenter prospective clinical studies.

## Introduction

Coronavirus disease 2019 (COVID-19) still continues to threaten the entire world. Globally, as of the beginning of August 2022, there have been 579.092.623 confirmed cases of COVID-19, including 6.407.556 deaths, reported to the World Health Organization (WHO) [1]. The COVID-19 pandemic, caused by severe acute respiratory syndrome coronavirus 2 (SARS-CoV-2), expanded rapidly throughout the world. The majority of patients (81%), have mild/moderate symptoms such as low-grade body temperature and cough. Patients with severe symptoms and critical cases make up only 14% and 5% of the population, respectively, but they might develop severe pneumonia, acute respiratory distress syndrome (ARDS), and multiple organ failure, necessitating hospitalization, and some of them may die [2-4]. Approximately 20% of the patients infected with SARS-CoV-2 develop potentially life-threatening pathologies involving hyperinflammation, cytokine storms, septic shock complications, coagulation disorders, and multiple organ failure. The mortality rate in the intensive care unit (ICU), where nearly 20% of hospitalized patients may need to be transferred, is approximately 61.5% [5,6]. There are some studies in the literature predicting the mortality of adult patients taking therapy in ICUs [7,8]. Early detection of risk factors for severe disease is crucial because there is currently no known cure for this illness. There are few publications on the clinical traits, incidence, and risk factors connected with the predictors of mortality, especially in patients receiving intensive care therapy, despite the fact that numerous articles have established the clinical features of COVID-19 patients so far [9,10]. Therefore, there is still a need for studies to predict mortality in COVID-19 patients using demographic and routine clinical laboratory test results.

This study was to create a novel diagnostic tool, the Biruni Index (Bir-In), which was connected with the likelihood of severe outcome and mortality in a positive quantitative real-time polymerase chain reaction (qRT-PCR)-confirmed COVID-19 patients admitted to a tertiary level care hospital's general ICU.

## Materials and methods

**Figure 1:**
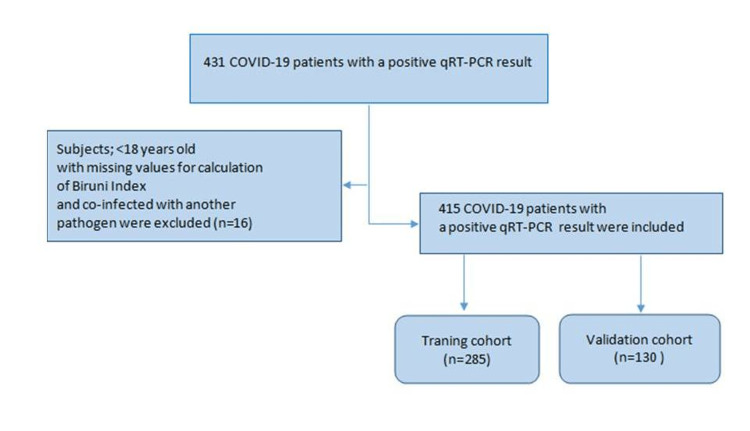
Flowchart describing the selection of study population qRT-PCR: quantitative real-time polymerase chain reaction.; COVID-19: coronavirus disease 2019

A total of 431 COVID-19 patients admitted to Biruni University Medical Faculty Hospital, Istanbul, Turkey, with COVID-19 pneumonia with positive qRT-PCR results between April 2020 and August 2021 were assessed for this retrospective study. The level of evidence for this cohort study is 2+ according to the Scottish Intercollegiate Guidelines Network (SIGN100) [11]. The local Ethics Committee of Biruni University, Istanbul, Turkey issued approval (approval number 2021-64-5). All patients involved in this study were diagnosed with COVID-19 according to WHO Interim Guidance 2020 [12]. A positive qRT-PCR result in the typical oropharyngeal swab or endotracheal specimens was required for inclusion. Being less than 18 years old, co-infection with concomitant fungal diseases, missing data for index calculation, and individuals co-infected with another pathogen were excluded (n=16). As a result, 415 COVID-19 patients with a positive qRT-PCR result were finally included in to the study (Figure 1). In the training cohort, variables associated with COVID-19-related mortality were determined using univariate logistic regression analysis, and then multivariate logistic regression analysis was performed to develop Bir-In. In the training cohort, variables associated with COVID-19-related mortality were determined using univariate logistic regression analysis, and then Bir-In was created using multivariate logistic regression analysis. In the training and validation cohorts, the diagnostic performances of Bir-In were evaluated by receiver operating characteristic (ROC) analysis. Since this index is a new invention, we did not have a chance to compare it with similar studies.

Clinical parameters

Body temperature, systolic and diastolic blood pressure, history of coronary artery disease (CAD), length of stay intubated, respiratory rate per minute, Glasgow Coma Score (GCS) (range: 3-15), sequential organ failure assessment, and diabetes mellitus (DM) were the clinical parameters used. 

Laboratory parameters

Complete blood count analysis (CBC), international normalized ratio (INR), glucose, aspartate transaminase (AST), alanine transaminase (ALT), D-dimer, urea, creatinine, lactate dehydrogenase (LDH), ferritin, C-reactive protein (CRP), procalcitonin (PCT), troponin I, triiodothyronine (T3), tetraiodothyronine (T4), thyroid stimulating hormone (TSH) levels, blood gas analysis, and a positive qRT-PCR result for COVID-19 infection were the laboratory parameters used. Using commercially available kits and blood samples taken on the first day in the general ICU, all of these laboratory parameters were assessed.

Statistical analysis

The statistical analyses were conducted using IBM SPSS Statistics for Windows, Version 23.0 (Released 2015; IBM Corp., Armonk, New York, United States). Using the Kolmogorov-Smirnov normality test, it was determined whether or not group distributions are Gaussian. When appropriate, continuous data were displayed as mean±standard deviation or median (25th-75th quartile). The Student's t-test or Mann Whitney U-test was used to compare continuous parameters between groups, and Pearson or Spearman's test was employed, for correlation analysis as appropriate. The categorical variables were compared using the chi-square test. The variables related to mortality caused by COVID-19 were identified using univariate logistic regression analysis. The Bir-In was created using multivariate logistic regression analysis to predict COVID-19-related mortality. To determine this index's diagnostic performance, ROC analysis was utilized. For the cut-off point of zero, the sensitivity, specificity, and positive and negative predictive values were determined. All reported *P*-values were two-tailed, and statistical significance was defined as P<0.05.

## Results

Features of the study population

A total of 415 COVID-19-positive patients with verified qRT-PCR were split into training cohorts (n= 285) and validation (n= 130). The demographics, clinical characteristics, and biochemical test results of the subjects are shown in Table 1 and Table 2.

**Table 1:** Demographic and clinical characteristics of patients with COVID-19 in training cohort BP: blood pressure; LDH: lactate dehydrogenase; AST: aspartate aminotransferase; ALT: alanine aminotransferase; WBC: white blood cell; NLR: neutrophil-to-lymphocyte ratio; PLR: platelet-to-lymphocyte ratio; CRP: C-reactive protein; COVID-19: coronavirus disease 2019

Variables	Non-ex (N=119)	Ex (N=166)	P-value
Age (year)	58±15	69±14	<0.001
Gender, n (F/M)	50/69	77/89	0.464
Biruni Index (Bir-In)	-1.15 (-2.22-0.07)	1.92 (0.63-4.00)	<0.001
Systolic BP (mmHg)	132±23	116±36	<0.001
Diastolic BP (mmHg)	66±11	61±16	0.003
Heart rate (beat/min)	85±12	96±20	<0.001
D-dimer (ng/mL)	872 (365-1902)	1783 (794-4877)	<0.001
pH (7.35-7.45)	7.44±0.07	7.37±0.16	<0.001
Urea (mg/dL)	36(28-54)	62 (42-104)	<0.001
Creatinine (mg/dL)	0.80 (0.70-0.93)	1.01 (0.76-1.73)	<0.001
LDH (U/L)	387 (295-540)	584 (402-770)	<0.001
Ferritin (µg/L)	420 (136-907)	819 (469-1687)	<0.001
AST (U/L)	37 (24-57)	43(32-69)	0.045
ALT (U/L)	35 (19-59)	33(24-60)	0.614
Body temperature (^o^C)	36.7±0.27	36.8±0.53	0.326
WBC (10^9^/L)	11.90 (7.53-20.07)	11.50 (8.03-17.22)	0.713
Platelet (10^9^/L)	286±103	254±108	0.090
Neutrophil (10^9^/L)	9.36 (5.37-17.12)	9.46 (6.72-14.57)	0.983
NLR	11.39 (4.91-22.02)	12.50 (5.60-26.06)	0.593
PLR	32.4 (17.5-74.7)	34.3 (11.6-59.1)	0.310
CRP (mg/L)	109 (41-158)	117 (49-163)	0.418
Procalcitonin (µg/L)	0.17 (0.08-0.54)	0.50 (0.25-2.44)	<0.001
Troponin I (ng/L)	11.8 (3.6-43.6)	38.5 (12.5-150)	0.002
Lactate (mmol/L)	1.6 (1.2-2.2)	1.9 (1.3-3.1)	0.032

**Table 2:** Demographic and clinical characteristics of patients with COVID-19 in validation cohort BP: blood pressure; LDH: lactate dehydrogenase; AST: aspartate aminotransferase; ALT: alanine aminotransferase; WBC: white blood cell; NLR: neutrophil-to-lymphocyte ratio; PLR: platelet-to-lymphocyte ratio; CRP: C-reactive protein; COVID-19: coronavirus disease 2019

Variables	Non-ex (N= 54)	Ex (N= 76)	P-value
Age (year)	62±16	68±14	0.025
Gender, n (F/M)	26/28	31/45	0.405
Biruni Index (Bir-In)	-0.80 (-1.80-0.69)	2.24 (1.00-3.66)	<0.001
Systolic BP (mmHg)	129 (117-141)	110 (89-148)	0.024
Diastolic BP (mmHg)	68 (55-72)	61 (49-71)	0.177
Heart rate (beat/min)	84±11	96±20	<0.001
D-dimer (ng/mL)	1051 (667-1628)	2126 (1012-6082)	<0.001
pH (7.35-7.45)	7.44±0.08	7.40±0.14	0.033
Urea (mg/dL)	46 (28-58)	78 (46-104)	<0.001
Creatinine (mg/dL)	0.78 (0.70-0.99)	0.96 (0.74-1.73)	0.002
LDH (U/L)	403 (290-658)	549 (374-782)	0.015
Ferritin (µg/L)	510 (108-1100)	956 (383-1658)	0.003
AST (U/L)	40 (20-66)	41 (26-61)	0.731
ALT (U/L)	32 (17-91)	36 (20-43)	0.960
Body temperature (^o^C)	36.8±0.55	36.8±0.52	0.719
WBC (10^9^/L)	9.41 (8.40-15.42)	10.73 (6.73-14.78)	0.591
Platelet (10^9^/L)	248±104	247±102	0.981
Neutrophil (10^9^/L)	7.11 (4.23-10.17)	9.83 (4.2-15.03)	0.224
NLR	12.40 (9.43-21.23)	12.25 (7.57-26.28)	0.913
PLR	33.6 (16.15-49.15)	37.92 (21.12-52.6)	0.460
CRP (mg/L)	109 (60-153)	123 (64-185)	0.502
Procalcitonin (µg/L)	0.185 (0.12-0.937)	1.13 (0.243-3.928)	0.022
Troponin I (ng/L)	10.6 (5.05-20.5)	26.6 (14.1-83.9)	0.003
Lactate (mmol/L)	1.6 (1.2-2)	1.8 (1.4-2.3)	0.257

Development of the Bir-In

In univariate logistic regression analysis, the training cohort's age, systolic and diastolic blood pressure, heart rate, respiration rate, blood pH, D-dimer, urea, ferritin, and LDH levels at first admission were statistically significant variables for the prediction of mortality. By using multivariate logistic regression analysis, Bir-In was created using all of these statistically significant parameters. Accordingly, Bir-In was constructed as following in Table 3; - 10.6 + (age * 0.05) + (heart rate * 0.04) + (admission breathe rate * 2.2) + (D-dimer * 0.0002) + (urea * 0.01) + (ferritin * 0.001) + (LDH * 0.003).

**Table 3:** Univariate and multivariate logistic regression analyses of variables associated with the COVID-19 related mortality LDH: lactate dehydrogenase; BP: blood pressure; COVID-19: coronavirus disease 2019

Variables	Univariate analysis			Multivariate analysis		
	OR (95% CI)	Wald	P-value	OR (95% CI)	Wald	P-value
Status of breath	7.683(4.070-14.502)	39.576	<0.001	9.116(3.955-21.014)	26.902	<0.001
Age (years)	1.055(1.036-1.075)	33.063	<0.001	1.054(1.027-1.081)	15.900	<0.001
Heart rate(/min)	1.038(1.021-1.054)	21.046	<0.001	1.043(1.020-1.067)	13.823	<0.001
LDH (U/L)	1.003(1.002-1.004)	23.473	<0.001	1.003(1.001-1.004)	13.719	<0.001
Ferritin (µg/L)	1.001(1.001-1.001)	21.983	<0.001	1.001(1.000-1.002)	9.827	0.002
D-Dimer (ng/mL)	1.000(1.000-1.000)	16.989	<0.001	1.000(1.000-1.000)	7.647	0.006
Urea (mg/dL)	1.020(1.011-1.028)	21.879	<0.001	1.011(1.001-1.021)	4.253	0.039
Systolic blood pressure (mm/Hg)	0.984(0.976-0.992)	15.870	<0.001			
Diastolic blood pressure (mm/Hg)	0.976(0.959-0.993)	7.564	0.006			
pH	0.010(0.001-0.091)	16.815	<0.001			

Bir-In has diagnostic usefulness for predicting mortality in training and validation cohorts. In both the training and validation cohorts, there were statistically significant differences for Bir-In between the groups (< 0.001 for both comparisons). The diagnostic performance of Bir-In was assessed by the area under ROC (AUROC) for the prediction of mortality in both cohorts. In both training and validation cohorts, Bir-In showed high accuracy for predicting mortality, AUROC of 0.901 (95CI%: 0.864-0.938) and 0.860 (95CI%: 0.795-0.926), respectively.

Diagnostic power of zero cut-off point of Bir-In for predicting mortality

In training and validation cohorts, accuracy, sensitivity, specificity, and positive and negative predictive values for Bir-In with a cut-off point of zero are shown in Table 4.

**Table 4:** Diagnostic utility of Biruni Index with a cut-off point of zero for predicting mortality in patients with COVID-19 infection AUROC: area under the receiver operating characteristic; COVID-19: coronavirus disease 2019; PPV: positive predictive value; NPV: negative predictive value

Parameters	AUROC (95CI%)	Accuracy (%)	Sensitivity (%)	Specivity (%)	PPV	NPV
Biruni Index	Training cohort					
		83.2	84.4	81.4	86.5	78.7
	Validation cohort					
		78.5	86.8	66.7	78.6	78.3

## Discussion

Following the emergence of the COVID-19 pandemic, many factors affecting mortality, especially in critically ill patients, have been studied. Among them, prognostic studies including vital parameters and biochemical markers during hospital admission are noteworthy [2,13]. Besides, prognostic scores with different characteristics were compared in critically ill patients during the COVID-19 pandemic [14-16].

The main research question of this study was to create a novel diagnostic tool to predict the mortality in COVID-19 patients with a positive qRT-PCR result admitted to the general ICU of a tertiary care hospital. For this purpose, Bir-In was created and validated in two different patient cohorts. It was concluded that Bir-In may be a useful prognostic index for any cause of mortality in critically ill patients with SARS-CoV2 infection. This research will contribute to the current literature to predict mortality from COVID-19 infection.

The usefulness of Bir-In in predicting severe cases and mortality of critically ill patients with COVID-19 on the first day of admission to the general ICU was investigated in this study. After adjusting for potential confounders in this new model, the risk of severe patients using COVID-19 to predict mortality was highly correlated with Bir-In and it was also a valuable predictor for mortality in patients with COVID-19 infection. Notably, it was found that the Bir-In, a reliable surrogate predictor of mortality, predicted severe cases and fatality in COVID-19 patients.

Therefore, a novel model, Bir-In, was developed for the prediction of mortality at admission with this study. Comorbidities have been established in various studies to play an essential influence on the prognosis of COVID-19 [7,8,10]. According to the results of this cohort study, respiratory failure, heart failure, an inflammatory storm, sepsis, disseminated intravascular coagulation, multiple organ failure, acute renal injury, and arrhythmia are all possible causes of mortality and other uncertain events. In this training cohort, age, hemodynamic and respiratory parameters such as systolic and diastolic blood pressure with heart rate, respiratory rate, pH, also blood urea, D-dimer, ferritin, and LDH levels were statistically significant clinical and laboratory parameters for the prediction of mortality. The results of our cohort study may be important in showing which prognostic parameters are statistically significant in critically ill patients.

In this retrospective cohort study, multiple risk factors for mortality in Turkish adult patients hospitalized with COVID-19 were discovered. Zhou et al. demonstrated multiple risk variables for adult mortality in Wuhan, China [2]. In their study, older age, higher D-dimer, and Sequential Organ Failure Assessment (SOFA) scores on admission were associated with the increased risk of in-hospital mortality. Furthermore, higher blood LDH levels were more common in patients with severe COVID-19. Ma et al. found that older age (60 years), followed by cardiovascular disease, hypertension, and diabetes, were independent risk factors for COVID-19-related mortality [17]. Age is an important indicator of severity, according to recent studies on COVID-19 infection and severe acute respiratory syndrome. In the current research, age, systolic blood pressure, diastolic blood pressure, heart rate, admission breathe rate, blood pH, D-dimer, urea, ferritin, and LDH levels on admission were identified as independent predictors of mortality in critically ill patients in the general ICU. In the training cohort, applying the zero cut-off point of Bir-In, accuracy, sensitivity, specificity, and positive and negative predictive values were 83.2%, 84.4%, 81.4%, 86.5%, and 78.7 respectively. In addition, in the training and validation cohorts, accuracy, sensitivity, specificity, and positive and negative predictive values for Bir-In with a cut-off point of zero are shown in Table 4. As a result, given the current pandemic situation and the significance of early identification of severe cases, Bir-In may be useful to clinicians. Because of the rising amount of healthcare spending in national economies in recent years, health-related cost-utility studies are gaining importance in almost all health systems around the world [18-20]. For these reasons, Bir-In, as a prognostic early diagnosis tool, can help in the early determination of optimal control strategies and cost-analysis in critically ill patients with COVID-19 infection.

During this short time period, many studies have been conducted in this field. Based on these studies, the predictors of mortality can be divided into five categories. These are demographics, clinical signs and symptoms, laboratory findings, imaging studies, and comorbidities. Among the demographic factors, age is one of the most important factors affecting mortality. In this study, age was significantly associated with increased mortality. Age-related abnormalities in immune cell function and increased production of inflammatory cytokines can be associated with increased mortality. Our study supported the findings of recent studies that have highlighted the role of clinical laboratory results in predicting mortality. For example, Tian et al. noted the role of LDH and CRP in increased mortality and they also found that troponin I, creatinine, and albumin levels were predictors of mortality in hospitalized COVID-19 patients [21].

In this retrospective cohort study, we included all COVID-19 patients hospitalized in our hospital for COVID-19 pneumonia since the outbreak began, and attempted to establish a mortality index based on medical biochemistry laboratory tests and clinical parameters that are routinely used in first-line healthcare settings. Bir-In, a novel model developed in this study, was successful in predicting mortality in critically ill patients with COVID-19 infection hospitalized for pneumonia. If confirmed by larger series of critically ill patients in prospective multicenter research, it may be more effective to predict the poor outcome for patients with COVID-19 infection. Age, heart rate, the status of breath, D-dimer, urea, ferritin, and LDH levels at first admission demonstrated an independent relationship with increased mortality. Besides, further studies may be needed to ascertain the relationship between possible risk factors with COVID-19 mortality.

Limitations of this study are being a single-center study, and not being conducted with a larger series of critically ill patients with COVID-19 infection of different races.

## Conclusions

The results of this retrospective cohort study were justified in the validation group, which may indicate that the Bir-In may be a useful prognostic index for any cause of death in critically ill patients with SARS-CoV2 infection. Critical care physicians can calculate Bir-In with patients' demographic and routine biochemical test results in a couple of minutes and can decide which patients need extra care. Besides, Bir-In can be helpful in designing of control strategies and cost-effectiveness analysis for critically ill patients with severe COVID-19 infection. However, these results should be supported by new prospective studies to enable the early identification of high-risk patients.
